# To start or not to start? An exploratory study of work meaningfulness among start-up co-founders

**DOI:** 10.3389/fpsyg.2024.1500036

**Published:** 2025-01-30

**Authors:** Deniz Dirik, Burak Özdoğan

**Affiliations:** Faculty of Economics and Administrative Sciences, Department of Business Administration, Manisa Celal Bayar University, Manisa, Türkiye

**Keywords:** entrepreneurial meaningfulness, meaningful work, meaningful entrepreneurship, significance, autonomy, cofounder identity

## Abstract

This study explores startup co-founders’ perceptions of meaningful work experiences in the dynamic and uncertain context of entrepreneurship. While entrepreneurial research has extensively examined firm-level outcomes such as performance and growth, the subjective experiences of entrepreneurs have remained relatively underexplored especially in an emerging country context. This study addresses meaningful work experiences of 12 startup co-founders from Turkiye by employing a qualitative research design and using in-depth interviews. Drawing on Self-Determination Theory (SDT) and the Job Characteristics Model (JCM), this research identifies key dimensions of meaningful work, including significance, autonomy, identity, challenge and resilience, recognition and support, and work-life balance. Our findings suggest that the complex interplay of intrinsic motivation and external validation contributes significantly to perceived entrepreneurial well-being and resilience. We present a three-dimensional model of perceived meaningfulness with professional, psychological, and societal aspects for meaningfulness experiences. We extend existing literature by demonstrating how startup ventures offer fertile ground for psychological fulfillment, not merely through financial success but through personal growth and societal impact.

## Highlights

- This paper explores startup co-founders’ perceptions of meaningfulness in their work, addressing meaning from an entrepreneurial lens.
- We introduce an entrepreneurial perspective on work meaningfulness, highlighting significance, autonomy, identity, challenge, resilience, and work-life balance, and offer a tripartite model incorporating professional, psychological and societal levels.
- Based on interviews with 12 startup co-founders, we seek to capture individual experiences of work meaningfulness.
- Beyond financial gain, we show how work meaningfulness fuels personal growth, resilience, and a sense of greater impact in the entrepreneurial world.

## 1 Introduction

In the vast expanse of entrepreneurial research, considerable attention has been devoted to exploring and explicating firm-level outcomes such as performance and growth (Wiklund et al., 2019) or economic and societal advantages such as poverty alleviation (Sutter et al., 2019). Although this body of work has advanced our understanding of the macro-level benefits of entrepreneurship, people pursue entrepreneurship for a variety of deeply personal and unique reasons. One such reason is the pursuit of meaning and purpose in one’s work and life in general, a fundamental part of human existence and quality of life (Frankl, 1959; Martela et al., 2021). There is still much need in the literature for studies into the subjective experiences of entrepreneurs themselves, particularly with regard to how they find meaning in their work in the unpredictable and volatile world of start-up ventures (Schwarz and Wahl, 2023). Additionally, previous research on meaningfulness has suggested that *“… the organizational task of helping people find meaning in their work is complex and profound, going far beyond the relative superficialities of satisfaction or engagement — and almost never related to one’s employer or manager,”* reflecting the relatively intrapersonal nature of meaningful work experience (Bailey and Madden, 2016). Addressing this gap, the present study seeks to explore the intricate psychological processes by which entrepreneurs navigate and construct work meaning amidst the inherent risks and challenges of entrepreneuring. We aim to explore the interplay between meaningful work and entrepreneurship, with a particular focus on the unique context of start-up ventures.

Understanding the psychology of entrepreneurs is crucial for grasping their motivations, behaviors, and overall well-being. Entrepreneurs often exhibit a higher degree of initiative and self-starting behavior compared to traditional employees, as they are known for their proactivity and ability to overcome barriers (Hahn et al., 2012). However, entrepreneurship is also rife with challenges such as exhaustion, work intensity, fatigue, and uncertainty, all of which impose, as de-energizing elements, significant emotional burdens on individuals and especially on founders (Kakatkar et al., 2021, 2024). Despite this, the emotional and psychological toll of entrepreneurship, particularly in relation to well-being and experiences of meaningfulness of co-founders, has received scant attention in both academic and practical discourse with some notable exceptions (Brieger et al., 2021; Kremer and Kruse, 2024; Schwarz and Wahl, 2023; Stephan, 2018).

Defined as an individual’s perception of *“… authentic connection between their work and a broader transcendent life purpose beyond the self”* and *“the subjective experience of how existentially significant and valuable people find their work to be”* meaningful work has been shown to be a critical factor for employee motivation, commitment, engagement, satisfaction, decreased turnover intentions and absenteeism, and higher performance, outranking other aspects of work such as pay, promotion or working conditions (Bailey and Madden, 2016; Martela et al., 2021). Overall, a meta-analysis has shown that people who find their work to be meaningful feel better and work better (Allan et al., 2019). Rooted in the understanding that work holds diverse meanings for individuals, previous research acknowledges the multifaceted nature of meaningful work, which extends beyond conventional metrics such as income and stability (Duffy and Dik, 2013; Shir et al., 2019). Such experiences of meaningful work and factors pertaining to promoting and constructing more meaningful experiences in organizations and a meaningful workplace have been vastly researched (Lysova et al., 2019) while the entrepreneurial perspective on meaningfulness has been comparatively underexplored. Entrepreneurs, especially those in start-up ventures, face a unique challenge; they must actively construct and foster a sense of meaning in their work, even in the face of extreme uncertainty, complexity, and ambiguity. This “meaning crisis,” in which entrepreneurs must generate their own sense of purpose amid volatile circumstances, is both a heavier burden and a potentially greater opportunity for personal fulfillment. This is particularly salient when we consider that employees often rely on their organization and leaders for deriving meaning from their work experience, founders have to create meaning by building and nurturing their own venture, which renders the latter’s experience much more personal and even an existential experience (Kakatkar et al., 2024).

The start-up environment’s capacity to propel individuals toward a deep sense of purpose and well-being (Shir and Ryff, 2022) makes the exploration of co-founders’ experiences of meaningfulness particularly relevant. Despite the inherent challenges of entrepreneurship, this environment offers rich possibilities for meaning making, and understanding this process is a timely and necessary endeavor. In this context, this study draws on two theoretical frameworks, namely Self-Determination Theory and Job Characteristics Model to probe the psychology of entrepreneurs, investigating how the start-up context fosters meaningfulness and contributes to entrepreneurial perceptions of meaningful work. To explore these dynamics, a qualitative research design was employed, utilizing in-depth interviews with startup co-founders. The study has focused on co-founders of start-up ventures to capture the richness and depth of their subjective experiences, the challenges they face, and the perceived meaningfulness derived from their entrepreneurial journey.

## 2 Theoretical background

### 2.1 Foundations of meaningful work

Self-Determination Theory has been used as a guiding framework in many studies exploring entrepreneurship (Al-Jubari et al., 2019; Du et al., 2021; Lanivich et al., 2021; Nikolaev et al., 2020). Self-Determination Theory offers a potent theoretical framework for explicating antecedents of meaningful work (Martela et al., 2021). The theory argues that humans have certain innate psychological needs, which are designated as autonomy, competence, and relatedness, and the satisfaction of those is a prerequisite for human wellness (mental and physical), vitality, growth, and sense of meaningfulness. From an entrepreneurial lens, SDT (Ryan and Deci, 2000) helps explain that meaningful work satisfies the intrinsic needs for autonomy, competence, and relatedness, enhancing motivation and engagement. A psychological theory of motivation that emphasizes the role of intrinsic and extrinsic motivation in human behavior and developed by Edward Deci and Richard Ryan in the 1980s, SDT suggests that people are naturally inclined to grow, develop, and engage in activities that foster psychological well-being when their innate psychological needs are met. The theory distinguishes between different types of motivation and explores how environmental and social factors influence motivation and well-being. Basic psychological needs at the core of SDT include autonomy, competence, and relatedness. Autonomy refers to the need to feel in control of one’s actions and decisions, and a sense of volition and willingness when engaging in activities, meaning individuals must feel they are acting in alignment with their values and interests (Deci and Ryan, 2008). Competence describes the need to feel effective and capable of achieving desired outcomes. This involves mastering tasks and gaining a sense of proficiency (Deci and Ryan, 1985). People are motivated to engage in activities where they can demonstrate their skills and capabilities. Finally, relatedness refers to the need to feel connected to others and experience a sense of belonging and attachment. Positive social relationships and feeling cared for by others are critical for fulfilling the need for relatedness (Ryan, 1995). Thus, autonomy is associated with a sense of volition and feelings of ownership for one’s actions; competence means having sense of mastery, efficacy and effectance in one’s work activities, and relatedness is the extent to which an individual feels connected to others, belonging and as part of caring relationships (Martela et al., 2021), all which have been shown to play key roles in evaluations of meaningfulness.

Studies have also utilized Job Characteristics Model to explore aspects of entrepreneurship (Rauch and Frese, 2007; Schjoedt, 2009). Job Characteristics Model (Hackman and Oldham, 1976), a framework used to understand how specific job characteristics affect employee motivation, satisfaction, and performance, posits that jobs can be designed to enhance intrinsic motivation and job satisfaction by incorporating key job dimensions. The model is widely used in organizational psychology and job design practices (Fried and Ferris, 1987). The JCM identifies five core dimensions of jobs that influence employee motivation, satisfaction, and performance, including skill variety, task identity, task significance, autonomy, and feedback. The JCM suggests that job dimensions such as task significance, identity, and autonomy contribute to perceiving work as meaningful. Accordingly, jobs that involve a variety of tasks are generally more motivating because they prevent monotony and allow employees to use different skills (skill variety). When a job involves completing a whole, identifiable piece of work from start to finish, an employee can see their contribution to the final product or outcome, and thus experience greater satisfaction (task identity). Employees are more motivated when they believe their work has a meaningful impact on others (task significance). Jobs with high autonomy foster greater responsibility and ownership of outcomes, which enhances motivation (autonomy). Feedback helps employees understand how well they are performing, which can improve motivation and learning (feedback).

Drawing from the premises of self-determination theory (Deci and Ryan, 2000) and job characteristics model (Hackman and Oldham, 1976) we posit that the satisfaction of fundamental needs for autonomy, competence, relatedness, identity, significance, and feedback plays a pivotal role in shaping the perceptions of meaningful work among individuals engaged in entrepreneurial activities. In the realm of start-up ventures, characterized by intrinsic motivation, self-directed behavior, and autonomous decision-making, the likelihood of perceiving work as meaningful is likely to be notably higher (Allan et al., 2016). Moreover, our study recognizes the potential of entrepreneurship, particularly within start-up ventures, to serve as a buffer against the challenges posed by the imminent waves of automation and the specter of the “useless class” (Harari, 2017). By understanding the intricacies of meaningful work in the context of start-up ventures, the research aims to offer insights into how individuals can find contentment even in the face of the bleak scenarios associated with human redundancy.

### 2.2 Meaningful work and meaning of work from an entrepreneurial perspective

Work meaning refers to individuals’ personal experiences and general beliefs, values, and attitudes about their work. When the practices of a work are aligned with social and cultural expectations, work is likely to be perceived as meaningful (Allan et al., 2019). As such, meaning of work is more about the type of meaning associated with one’s work, while meaningful work is more about the amount of significance attached to one’s work (Rosso et al., 2010). Although generally used in an interchangeable way, meaning of work and work meaningfulness are not necessarily synonymous concepts with the latter denoting an inevitably positive meaning. Much of the research in the meaning of work literature uses meaning of work or work meaning in a positive lens, implying a work meaningfulness perspective. Rosso and colleagues argue that “meaningful” and “meaningfulness” would be more accurate choices when authors of meaning research address the amount of significance individuals associate with their work, and we follow their lead.

From an organizational viewpoint, the meaning of work has been embedded primarily in a psychological perspective with an emphasis on subjective interpretations of work experiences whereas a sociological perspective would underline the role of social or cultural value systems in ascriptions of meaningfulness to work activities. The extant research in organizational behavior has emphasized the psychological perspective of individual experiences and feelings rather than societal forces in understanding work meaningfulness. The meaningful work in that sense enables feelings of transcendence, encourages identification with one’s organization and purpose, and helps promote positive organizational outcomes (Jiang and Johnson, 2018; Pratt and Ashforth, 2003; Rosso et al., 2010). As such, meaningful work provides benefits across individual and organizational levels, making meaningful work a priority for both employers looking to boost their organization’s performance and individuals for eudaimanic wellbeing and higher satisfaction with life (Johnson and Jiang, 2017; Schwarz and Wahl, 2023).

Although a variety of factors have been identified as potential sources of meaning such as individual attitudes or organizational values with little consensus on a general framework, work meaningfulness has emerged as a pivotal construct in understanding organizational outcomes such as employee engagement, satisfaction, and organizational commitment (Rosso et al., 2010). Because of such benefits, meaningful work and experiences/activities that spur it has received considerable attention in the literature (Both-Nwabuwe et al., 2017; Jiang and Johnson, 2018). Drawing on interviews with 135 individuals from 10 different work domains, including entrepreneurs, a previous research study defined meaningful work as individuals’ perception of some authentic connection between their work and a broader transcendent life purpose beyond their self (Bailey and Madden, 2017). This study found that individuals perceiving their job to be fulfilling of their potential, creative, absorbing, and interesting are more likely to perceive their work as meaningful as well as those who receive recognition for their accomplishment, and that meaningful work does not always imply positive work experiences. Steger et al. (2012) define meaningful work, a multidimensional construct, as “*subjectively meaningful experience consisting of experiencing positive meaning in work, sensing that work is a key avenue for making meaning, and perceiving one’s work to benefit some greater good*.” The significance of finding meaning in one’s work transcends mere economic necessity, encapsulating a desire for purpose, value, and impact (Schnell and Hoffmann, 2020). Work meaningfulness is defined as the subjective sense that one’s work is significant, purposeful, and contributes to a broader spectrum. It encompasses individuals’ perceptions of their work’s value beyond its tangible rewards. Meaningful work literature offers various elements of meaningfulness such as significance, broader purpose, and self-realization (Martela and Pessi, 2018) or significance, purpose, coherence and belonging (Schnell and Hoffmann, 2020; Schwarz and Wahl, 2023). Meaningful work and research on how to find meaning has been receiving attention from practitioners probably because a lack of meaning can have negative and even severe consequences such as absenteeism or depression. The meaning of work has been associated with many important outcomes in organizational settings such as work motivation, engagement, job satisfaction, empowerment, organizational identification, career development, individual performance, and personal fulfillment as well as associations with negative outcomes such as stress and absenteeism (Glavas and Kelley, 2014; Lepisto and Pratt, 2017; Pratt and Ashforth, 2003; Rosso et al., 2010; Steger, 2016).

Although research has long acknowledged that entrepreneurs undergo extreme emotional experiences in their daily initiatives and that their well-being may be compromised on a daily basis few studies have examined the role of meaningfulness and psychology of meaning behind entrepreneurs’ actions. With this study, we address the role of perceived meaning for startup co-founders. Previous research has ascribed the term “the new money” for meaningful work, implying the financial and performance benefits that accompany perceptions of meaning and purpose (Schwarz and Wahl, 2023).

In an interview with his brother, the founder and CEO of OpenAI Sam Altman refers to founder burnout as a product of things not working and a lack of momentum (Combinator, 2016). And in another address to the question of how to deal with burnout as a founder, he offers a stark yet motivating perspective: “*it sucks and you keep going. Unlike a student where you can sort of throw your hands and say, you know what, I’m really burned out. I’m just going to get bad grades this quarter. One of the hard parts about running a startup is that it’s real life and you just have to get through it*.” (PSkinnerTech, 2023). This statement encapsulates the formidable pressures faced by founders, who often carry the weight of their ventures on their shoulders, with scant room for personal setbacks. In the dynamic world of startups, co-founders are frequently caught in the tension between fostering value and the imperative of generating profit. This dichotomy not only challenges their resilience but can also veer them away from their foundational goals, with repercussions on the perceived meaningfulness of their work. The pursuit of balancing societal contribution with profitability poses a complex dilemma, occasionally diverting them from their initial mission, with significant implications for their sense of purpose and the broader impact of their venture.

Entrepreneurship has been characterized as an emotionally demanding and uncertain journey associated with high levels of stress and fear in addition to comparatively higher levels of job satisfaction (Shir et al., 2019). Although entrepreneurial ventures come with high levels of uncertainty, risk, potential for bankruptcy, and many other challenges coupled with high levels of stress and anxiety, many entrepreneurs report being satisfied with their lives (Schwarz and Wahl, 2023; Stephan, 2018). To what extent such feelings of satisfaction correlate with entrepreneurial search for and creation of meaning and purposefulness remains to be explored. Additionally, research distinguishing between outcomes of necessity versus opportunity entrepreneurship finds that the former correlates with lower educational attainments, lower levels of wellbeing and life satisfaction, higher financial concerns related with debt accumulation, and lower levels of autonomy and growth opportunities. Opportunity entrepreneurs driven by a growth and independence mindset might thus be more likely to seek and find meaning in their pursuits. Opportunity entrepreneurs, assuming an ideal and virtuous kind of entrepreneurship in contrast to a vicious and malevolent counterpart (Ryff, 2019), are more likely to transcend a pursuit of self-gratification to consider the wellbeing of others such as employees, the community in which their ventures are embedded, the environment as well as the society and the planet at large. This provides the basic rationale for our choice to interview startup co-founders.

Previous research has investigated the relationship between meaningful work and entrepreneurship, comparing the perceived meaningfulness of work among entrepreneurs, employees, and freelancers (Kooskora and Vilumets, 2020), highlighting that entrepreneurs tend to report higher levels of perceived meaning in their work, particularly in dimensions such as inspiration, unity with others, and self-development. That is, entrepreneurs experience higher levels of meaning in their work compared to employees, especially in areas that align with personal growth. A large-scale research study has confirmed that self-employed individuals are more satisfied with their lives than paid employees even when compared within similar occupations (Hessels et al., 2018) and higher job satisfaction associates with jobs that are high in autonomy, task variety, and task significance (Hytti et al., 2013). A critical factor that relates to entrepreneurs’ well-being is the extent to which they find their work meaningful, whether they can make a genuine difference with their business activities. Additionally, entrepreneurs who create social value experience heightened self-esteem and self-worth by aligning their business activities with societal benefits. This alignment strengthens their positive self-concept, which enhances their job satisfaction and reduces burnout (Brieger et al., 2021). While research has explored meaningful work experiences in the context of specific industries or individual topics (Schwarz and Wahl, 2023), this paper examines the extent of meaningful work experiences and the “meaning compass” of startup co-founders.

## 3 Method

Given the exploratory nature of our study, we chose a qualitative research design incorporating in-depth interviews with start-up co-founders to allow for a rich exploration of their experiences and perceptions. This methodology aligns with our intention to capture the subjective realities of entrepreneurs, providing valuable insights into the meaningful work and entrepreneurship nexus. Qualitative research explores a problem or topic by identifying variables that are not easily measurable (Creswell and Poth, 2016). Instead of establishing cause-and-effect between variables of interest, the underlying motivation in qualitative approach is to observe data related to the context, processes, and perceptions. This approach enables an observation of patterns and understanding of the perspectives of actors within their specific contexts (Glesne and Peshkin, 1992). Through our study, we hope to be able to respond to calls for more qualitative research into entrepreneurship (Van Burg et al., 2020) which helps advance our understanding of entrepreneurship as a phenomenon (Suddaby et al., 2015). In sum, the research design centered on salient experiences of co-founders and their perceptions of meaning regarding their entrepreneurial actions. The number of qualitative studies within entrepreneurship research is on the rise thanks to its capacity to make meaningful contributions to the field (Javadian et al., 2020).

### 3.1 Data collection

We adopted a purposive sampling approach to identify and recruit participants, focusing on co-founders of start-up ventures. Our recruitment began on LinkedIn, where we contacted a previous connection who fit the study’s criteria. Following this initial contact, we employed a snowball sampling strategy, leveraging the networks of each participant to identify additional potential participants. This iterative process allowed us to build a broader pool of self-identified co-founders, enhancing the diversity of our sample.

In total, we reached out to 87 co-founders through LinkedIn. Of these, 42 responded to our inquiry: 25 declined participation, while 17 consented to be interviewed. During the interview process, three participants were unavailable for any of the scheduling options provided over a two-month period. Additionally, two interviews were excluded from the analysis: one participant later withdrew their consent, and another, who transitioned to a traditional white-collar career, was removed based on inter-rater agreement due to concerns about the relevance of their perspective at the time of the interview. These considerations resulted in a final sample of 12 co-founders whose interviews form the basis of our analysis. This sample size aligns with qualitative research standards, ensuring both depth and diversity of insights while achieving thematic saturation (Creswell and Poth, 2016; Glesne and Peshkin, 1992).

Based on the theoretical framework, we try to understand how co-founders of start-up ventures describe the meaningfulness of their work, and how their perceived meaningfulness helps them tackle work-related challenges. Through an in-depth exploration of startup co-founders’ firsthand experiences, we also seek to identify what role psychological experiences such as autonomy, significance, identity, and social support play in shaping the meaningful work experiences of start-up co-founders, and how challenges and uncertainties inherent in start-up ventures influence entrepreneurs’ perceptions of meaningful work and their overall well-being. Relying on these overarching questions, we have identified a set of questions as guideposts during the interviews. For interviewing co-founders of start-up ventures on the topic of perceived work meaningfulness, we crafted questions to capture the richness and depth of their experiences, challenges, and the meaningfulness they derive from their entrepreneurial journey. These questions have been designed to elicit insights into the co-founders’ perceptions of meaningful work, their motivations, challenges, and the broader impact of their start-up. Sample questions include, *What inspired the vision and mission behind your start-up?, Can you share a moment or experience in your start-up journey that you found particularly meaningful?, How do your personal values align with the goals and practices of your start-up?, Can you describe a challenging time in your start-up and how you found meaning or growth in that experience? and How do you believe your start-up contributes to societal well-being or addresses social challenges?*

### 3.2 Interviews and data

Co-founders are the focus of interest in our qualitative study. A total 12 start-up co-founders from Turkiye have participated in our study (see Table 1). We approached each one of them and collected data through personal interviews to enable an in-depth exploration of the topic. Individual semi-structured, open-ended interviews were conducted on Zoom, with each session lasting approximately 1 h. The interview protocol began with general questions to establish rapport with the participants. On average, each co-founder interview transcript is 18 pages. These questions focused on the participants’ experiences, feelings, beliefs, and convictions regarding their entrepreneurial ventures. Both planned and impromptu questions were used to elicit further details. Each interview was audio-recorded with the participant’s permission and transcribed verbatim. We used an AI program to transcribe the interview records. We also undertook steps such as reviewing archival online data, organizational websites, newspaper articles, and conducting LinkedIn searches to triangulate and validate interviewee responses, particularly for fact-checking purposes related to startup ventures and co-founders. This process also allowed us to familiarize ourselves with the interviewees prior to the meetings.

**Table 1:** Participant demographics.

Participant ID	Gender	Position	Started in	*N* of people working	Industry
F1	Female	Cofounder	2018	Between 2 and 5	Biomedical equipment and software
F2	Male	Cofounder	2021	15	Artificial intelligence
F3	Male	Cofounder	2022	4	Energy/clean electricity production
F4	Male	Founder/owner	2022	22	Virtual/augmented reality
F5	Female	Cofounder	2021	8	Renewable energy/hydrogen
F6	Male	Founder	2021	12	Digital marketing/dynamic location advertising
F7	Female	Cofounder/CEO	2020	25	Energy benchmarking
F8	Male	Founder	2017	17	Hospitality industry software development solutions
F9	Male	Cofounder	2017	40	Digital marketing
F10	Female	Founder/owner	2021	Project-based temporary teams	Consultancy
F11	Male	Cofounder	2019	5	Cyber security
F12	Female	Cofounder	2021	5–20	Biotechnology

### 3.3 Analysis and thematic coding

We followed an iterative approach in our thematic coding, beginning with open coding to identify first-order concepts, followed by axial coding to develop overarching themes. Discrepancies in coding were resolved through collaborative discussions among the authors. We adopted traditional content analysis and undertook an iterative process of reading and categorizing participants’ statements to create our first-order codes. The finalization of these first-order codes took multiple waves of iteration with continuous discussions between the authors and through the resolution of disagreements. We went back and forth between the data to (1) discover and narrow, and (2) to enrich and validate (Mathias et al., 2015). Reading interview transcripts multiple times, we looked for and began to realize certain commonalities across responses and referred to the existing literature to create themes. This enabled us to link our preliminary findings back to theory and build connections with the extant literature. However, we did not aim to confine our coding only to the theoretical mapping or predefined constructs.

Two researchers coded the data with frequent meetings to resolve coding conflicts in order to reduce the possibility of bias in the interpretation of qualitative data. Systematic coding and analysis were used to increase reliability. This initial stage aimed to identify recurring concepts and ideas grounded in the participants’ language. Each author independently reviewed the transcribed data, marking phrases, sentences, or short paragraphs with relevant or potential codes and categories. At this stage, we utilized a conventional content analysis approach, allowing codes to emerge inductively from the data (Senthanar et al., 2021). For instance, when participants discussed challenges related to “work-life balance” or described experiences of “resilience,” these phrases were coded directly as such. After the initial coding, we engaged in a collaborative process to compare and refine the codes. Emerging themes were then mapped onto the theoretical premises of Self-Determination Theory and the Job Characteristics Model (Deci and Ryan, 2008; Hackman and Oldham, 1976). This iterative process involved comparing inductively derived codes with the deductive insights drawn from meaningfulness literature, particularly regarding autonomy, competence, relatedness (SDT), and job characteristics like significance, task variety, and feedback (JCM). This combined inductive-deductive approach ensured an alignment between participants’ lived experiences and the theoretical frameworks underpinning the study. For example, participants’ references to managing flexibility while balancing family and work were coded under work-life balance through inductive coding. These codes were further contextualized within the autonomy and resilience dimensions of SDT, demonstrating how balancing demands enhances perceived control and coping capacity, through deductive relevance.

The iterative coding process continued until consensus was reached, resulting in a thematic structure that integrates data-driven insights with theoretical constructs. This approach enabled us to build a multidimensional model of entrepreneurial meaningfulness that reflects both the unique nuances of participant experiences and the broader theoretical findings of meaningful work literature.

## 4 Findings

Based on the interview data, we identified Dimensions of Work Meaningfulness for Start-up Ventures. As such, the paper proposes a multidimensional model of work meaningfulness drawing mainly from Self-Determination Theory and Job Characteristics Model which state that satisfaction of the intrinsic needs for autonomy, competence, and relatedness as well as skill and task variety, significance and feedback are critical for human wellbeing (Deci and Ryan, 2000; Hackman and Oldham, 1975), and these in turn contribute to perceptions of meaning at work, enhancing motivation and engagement.

### 4.1 Significance

Significance is the belief that one’s work matters and has a positive impact on oneself, others, or society. Finding significance in one’s work goes together with the belief that you have an impact which is positive (a negative impact is also possible, and it would not be associated with meaning-making in the sense we imply in this paper). Significance is also associated with opportunities for growth. Some participants characterize “significance” with creating value first for themselves to provide a means for self-perpetuation and then using this as leverage to create significance in a more altruistic way, that is for people, environment, and society at large. Participants also assume different opinions and ideas of the concept “significance” when asked what they find to be significant or what provides them with a sense of significance about their work, product, idea, or model. The opportunity to live a decent life through one’s entrepreneurial initiative is considered a means of finding significance as is the possibility to realize one’s ambitions and passion. Trendsetting, game-changing and original ideas act as perpetrators of significance and are proudly mentioned by participants. Participants claim that what provides them with significance also offers a window to satisfaction with one’s job, motivation, and opportunity to make a difference. Significance helps them get rid of chronic or existential boredom and find value in what they do both at and after work. These ideas do however seem to be influenced by and change through time, culture, and context. Sample quotes from interviews are presented in Table 2.

**Table 2:** Quotes concerning the category “significance.”

Codes	Quotes
Positive personal impact	*“Well, in the end.. No matter how much benefit we provide.. Even if this is satisfying for a person... At the end of the day, there’s money in this. Without making money...We cannot fully find our motivation.” (F3)* *“Now, yes, let the customer be satisfied, but I also want to issue invoices already. I mean, I want to see their satisfaction reflected in the invoice as well. Things are starting to become more tangible with metrics.” (F6)* *“The fundamental purpose of all startups is this. If we set aside some social impact ventures, the basic math behind all startups is essentially, ‘Let me solve a problem, sell this solution, and make money in return.’ At the end of the day, we also founded X to make money, of course. I really love the work I’m doing or the thing I’m creating. But at the end of the day, I need to sell this business at some point. I need to exit at some point. So, for me, this is a bit more like this: When I think pragmatically, I have a goal. I can say that I’m on the path to that goal with X.” (F11)*
Positive social impact	*“Well, I guess its place in my life was that I had a concern, and this was a way to express it. I mean, these concerns were missions I took on because of my job and my character, like showing more respect for nature, being environmentally conscious, valuing women, and promoting respect for nature...That’s why playing with the dynamics both leads to being excluded from society and keeps you labeled as an activist because you are somewhat rebelling against the big players. But I guess this also fuels us. We have a sustainability manifesto. We strive not to work with institutions and projects that do not adhere to this manifesto. We’ve succeeded in this for 3 years. I hope we can continue for another 3 years without straying from it.” (F5)* *“For example, as X, we reduced carbon emissions by 220,000 tons. We planted the equivalent of 10 million trees. We achieved $35 million in cost savings. And we are making the world greener and more livable. That’s actually one of the things that keeps us going—being more beneficial to the world. The second is being beneficial to the country. I’ve always had strong patriotic feelings about supporting local and national efforts.”* (F7) *“The concept of personal data is something very new in Turkey, and we produce a lot of content and organize many events to push people towards understanding it. We try to explain that personal data is valuable. In today’s world, data is like oil. In the past, coal was needed to run factories, and coal was obtained in* var*ious ways—through colonization, etc. Now, data is the fuel for companies like Google, Facebook, TikTok, Amazon, and these companies make money from it. We also try to emphasize that data is something very valuable, that user consent is crucial, and that personal data is a personal right—meaning it should not be violated. We convey this strongly to both companies and individuals. That’s why I think it has a societal impact because people are now questioning, ‘Where are you sharing my data?’” (F11)*
Opportunity for growth and self-expression	*“Our goal is this. No person should be forced to do the same job over and over again. There are things that being human makes us realize or grants us. The biggest one of these, the only thing that separates us from other creatures, from animals, is that we can create something out of nothing by using our creative power. And every person should be able to experience this.. Every country should be able to provide a minimum wage as unemployment benefits to everyone who is not working, at least to a level where they can survive. All remaining, ongoing, repetitive tasks need to be taken over by either artificial intelligence or other technologies. We are saying that people should be able to move toward more creative professions, toward things like art where they can find themselves. With the latest developments, this might not be a very realistic scenario in the near future.” (F4)* *“This, actually, as you know, when working in institutions—whether it’s a university or a ministry—there’s a daily calendar routine you have to follow, and we have many stories about this. After a while, when you get into this daily repetitive cycle, you start asking yourself, ‘What am I doing? What am I contributing to?’ It becomes just about following a calendar on cold days. I work in the evenings, I work on weekends, to meet the needs of an academic or an entrepreneur. In fact, while working there, I encountered certain obstacles. I could not do most of the things I wanted to. That’s when I thought, ‘I can do this independently.’ And that’s how I made my exit from there.” (F10)*
Making a difference	*“But at the same time, I mean, touching people’s lives is, yes, a satisfaction in my opinion. But what truly satisfies me is this, actually, I mean, it feels a bit like a game to me at some point. I mean, we are constantly trying things, like we change the sentences we use, and people enjoy it.” (F1)* *“We’re all going to die in the end. We have limited time. I want to make sure that I make a difference between the time I arrived and the time I leave.” (F7)*
Value added	*“I mean, doing something with added value. Because these kinds of things excite me. If we are doing a job, I believe we are motivated as human beings when we are doing something that can provide added value. This motivated me because making money is a different story. There are many different ways to do that. If I had entered this just to make money, I would not have done this job; I would have been a civil engineer…I think we are filling this gap with the X mobile application side. Because we are applying personalization technology there. People can choose their mood. They can say, ‘I want to watch a game today,’ or ‘I’m feeling down today.’ They can say, ‘We’re a group of six today.’ Our algorithm already recognizes them. It recommends the most suitable venue where they can be happiest in their location. Beyond that, we have people with diabetes, people with hypertension, people with celiac disease. We have many citizens who have reactions to various allergen groups. I’m talking about millions of citizens. We are also partnering with the Istanbul Metropolitan Municipality (İBB) right now. İBB has an application used by 3 million people monthly. We are recommending venues to all Istanbulites there. While doing this, we consider both personalization and their individual restrictions. For example, we notify someone with diabetes of what they should avoid consuming on the menu.” (F8)* *“At the point we have reached, our users have spent over 1 million hours, or 104 years, interacting with our smart menus. From day one, we have been very conscious about working with this data, and our AI model has reached a point where it tells businesses to increase the price of this product, lower the price of that one, remove this product from the menu, and highlight these products during certain time frames, increasing their revenue by up to 30%. Honestly, I’d say the most important factor here is the added value because that’s the part that brings people happiness, and I think that’s the crucial part…I always think about what kind of added value third parties we work with can bring us, just as I do in my own work.” (F8)*
Trendsetting and original ideas	*“The thing there, again, was about changing a rule, changing the game, doing something differently. I mean, this job could have been done remotely. Then I strongly opposed the pricing model. Everyone was offering fixed prices, commissions, percentages, etc. I said, ‘Look, I have an hourly rate, and I’m going to issue invoices based on human hours.’ That was something that did not exist in Turkey at the time. Now, all agencies have shifted to that model. Changing the game a little bit was very motivating for me. And now, what really motivates me is knowing that something better can be done than what I’ve already done.” (F9)*

### 4.2 Autonomy

Autonomy represents the degree to which individuals feel they have control over their work processes and decisions. Autonomy does not mean a lack of constraints or limits nor absolute freedom but rather a perceived independence and capacity to make an impact without having to get permission from someone else or having to do so within some limits because of power imbalances (such as financial dependence on investor/s or based on political/cultural/organizational barriers). Participants report benefiting from flexible decision-making processes and delegation of responsibility to team members based on expertise and trust. Autonomy in this sense is achieved through working with people who are not “colleagues” but “team members.” Some participants report having created a work culture where their absence is happily compensated by team members and moral licensing allows them to feel okay with a tad of otherwise irresponsible behavior, such as not feeling motivated to go to the office one fine morning and staying at home. Autonomy is a mindset where there is space and tolerance for individual preferences, expectations, and problems. Startup co-founders declare being content with both their own autonomy and providing their teams with greater autonomy and space. However, it is also the case that autonomy might be the least of concerns when there are financial or legal challenges to deal with or when they experience a crisis with customers, consumers, investors, or team members. Autonomy is a concern for moments when things are on track. Sample quotes are presented in Table 3.

**Table 3:** Quotes concerning the category “autonomy.”

Codes	Quotes
Independent decision making	*“First of all, I am independent, and I really try to do things the way I want to. Since there’s such a broad space where I can do what I believe is right without being bound by an obstacle, a different way of thinking, or a school of thought, I feel very free. In a way, the meaning of this work for me is also freedom, and somehow, it’s still related to serving others.” (F10)*
Flexibility instead of strict hierarchical structuring	*“Sure, I do not necessarily work 8 h a day. I probably work more, but I can choose my own working hours. That’s something really beneficial for me. Because, for instance, the companies we are working with on a contract basis, one is based in Switzerland and the other in the U.S. So, our hours are all over the place. Sometimes we need to have meetings at 2 or 3 in the morning.” (F4)* *“I give them all a lot of space. I always allow them to make mistakes. Because I think personal development is something like that.” (F7)* *“There has never been a process at the company where it’s like, ‘nothing happens without me.’ There’s no such story. But they are already polishing their ideas thanks to us.” (F8)*
Delegation of responsibility to the experts in the team	*“In certain matters, in certain departments, it’s very important that I no longer have the final say and that they are the ones telling me what’s right. We’re seeing this in the team right now. Some of our colleagues have embraced their positions and departments so much that they can say, ‘This is my area. You may be the founder of the company, but I will be the one to say what’s right here.’ And that’s a really great thing…I think that’s a great freedom. Yes, we work very hard for this. But, for example, if there’s a change in the business idea, a change in the product, or we see a change in society or our target market, we can immediately reflect it in the product and bring it to them. It’s very exciting to be able to do that. Despite all the exhaustion, it’s very fulfilling. Secondly, no matter how long the hours we work on this, it feels like this is just how it’s supposed to be—like this is my nature...I feel very, very free. I mean, any work I do here—like yesterday, for instance, I had about eight meetings back-to-back. Most of them were with international investors, and some were internal team meetings. Yes, it gets very tiring, but it never makes me feel like, ‘Oh, I have eight meetings today, and so many hours have passed.’ For me, it feels more like a way of breathing—it’s good. At least for now, it feels that way. That’s why I feel free.” (F2)* *“That’s why my work freedom is very high. I’ve learned delegation during this process. The things a CEO should and should not do. I try not to get too hands-on with tasks, and I try not to micromanage. That’s why we have always aimed to hire self-motivated people when selecting the team. Right now, everyone is aware of their own work and responsibilities; they assign their own tasks and close them themselves. I can say that at the end of the day, I just make sure this system works smoothly. That’s why I’ve felt very free and comfortable over the last 6 months.” (F11)*
Trustful relationships	*“Right now, I can say that we are completely free. At this point, we are just in the phase of having things approved and verified by each other.” (F1)* *“Well, at least I feel like I have people around me that I trust. That kind of puts me in a good place. Because, for example, I do not know anything about the accounting side, or I do not know anything about the legal side. I could be getting scammed right now in the simplest way, and I would not even realize it. But at least I have trusted people around me. I know they are doing the right job. I will not have any problems.” (F4)* *“But between the four partners, there’s a partnership agreement. We really spent hours thinking, ‘If this clause is included, I will feel secure. But if that one is, I will not. So, what’s the common ground?’ We handled those parts very transparently. While we may not be as transparent in the financial aspects, we are very transparent in these areas. And, we are also transparent in the financial aspect in this way: while the debt burden is on me, we are transparent about revenue sharing and income even with the team. So, I think we are free because of our transparency.” (F5)*
Moral licensing	*“Just yesterday morning, I woke up. Took a shower. Got ready to leave the house and said, ‘I do not want to do anything today. I do not want to do anything today.’ I called the team and said, ‘I’m not coming in today.” And they said OK... We already manage each other like this. Maybe thanks to having partners. If I were alone, I would not be able to do this.” (F3)*
Providing space	*“I do not interfere with what the development team is doing because the development team works with the product team. The product team meets with customers. There’s something that comes out of everyone touching on each other’s work and using collective intelligence. And believe me, most of the time, I see the result when it goes live. I used to fight a lot. I would say, ‘You’re not leaving me any room to play. You made me the CEO, but I only hear about everything at the end.’ But over time, I realized that this is the right way. Not just the right way, but the beautiful way.” (F9)*

### 4.3 Identity

Identity refers to the alignment between one’s work and personal values, beliefs, and sense of self. Some participants tend to define their business as part of their body like a limb or sometimes like a child, adopting parental terminology. The lines between the person’s character and the business gets blurred. For example, if co-founder has a large risk appetite, this reflects on the startup itself through a spillover effect. The startup, its birth, its life cycle, its potential demise, and significant turning points are described as critical life experiences in co-founder’s personal life. There are descriptions of feelings of excitement about work-related experiences in a way when one falls in love or feelings of utter disappointment, disillusionment, misery and even depression in case of setbacks, roadblocks, and failures. On the other, some participants demystify such narratives by positioning their entrepreneurship in a more pragmatist framework and distance their self from their current business. Their identity is more aligned with “entrepreneurship” as a professional process and less with whatever they are on at the present. There are also stories that connect the entrepreneurial journey to a personal narrative in the founder’s life experience. From an identity perspective, startup initiative contributes to exploring, realizing, reflecting, developing, and transforming one’s inner self, mentality, mindset, and world view. Sample quotes are presented in Table 4.

**Table 4:** Quotes concerning the category “identity.”

Codes	Quotes
Self-work nexus or becoming one with work	*“I think for many entrepreneurs, startups almost become like a second identity. And I realized that the boundaries of the startup are where our own limits end.” (F1)* *“At that point, if I did not associate X with myself, I would feel very trapped. But right now, since X’s growth directly corresponds to my own personal growth, I feel freer than I probably should. A startup is truly something that is identical to its founder, in my opinion. Certain values in your way of thinking somehow move it forward.” (F7)*
Startup as one’s baby or pragmatist lens	*“I think I’ve passed the hard part a bit—the survival and the ‘giving birth to the child’ phase. Now, hopefully, we’ll move on to the growing phase.” (F6)* *“For me, its meaning is this. I’ve never been the kind of person who thinks, ‘This job is my baby, my life.’ It’s not a love or a blind devotion. I approach it in a much more pragmatic way. For me, X is like a field battle right now. I’m like a football player, I’m on the field. And once X reaches success, once we become champions, I’ll make my retirement and move to the stands. After an exit or reaching a point of success, I’ll start taking on different identities.” (F11)*
Critical life experiences	*“But doing your own thing and being able to reach this point really makes you feel good. It makes you feel confident…That’s why this is my last shot. If I cannot make this one, I do not know what will happen. So, we are all giving everything we have got.” (F3)*
Values and beliefs	*“So, I think if there’s someone who can do this job in Turkey, it’s me. What keeps me going, actually, is my belief in the job. My belief in the job and in myself. That’s why I have not considered any other job. I mean, it’s very clear when they say part-time entrepreneurship does not work. Right now, I’m looking at positions in our company, and I’m trying to eliminate part-time roles, no matter the level. It should not be part-time. That’s kind of a dilemma too. You’re putting 100% into this, putting all your eggs in one basket.” (F6)*
World view and life philosophy	*“Entrepreneurship is actually a philosophical subject. It’s not something that can be easily explained. In the end, the concepts of entrepreneurship and startups have also started to diverge. While starting your own business is entrepreneurship, a startup is about being able to create a digital entity, a world—these are different topics. When we look at really successful startups, we always see this: they have a philosophy. The founder has a philosophy, and the company has a philosophy. For example, for a long time, what Google sold was ‘do not be evil.’ It wasn’t being the evil company within Silicon Valley or the capitalist system of America. The lines it drew for its employees, the way it attracted people, were always discussed. The best, the top people always wanted to go to Google. And there, they created a culture, a philosophy. Now, in Turkey, we are seeing this again in different companies. It has to be an attraction point. It has to be able to establish its own culture from the very beginning. It has to be able to establish its philosophy. That’s why I’d say the secret to a successful startup is parallel with the company’s philosophy.. Because it’s a job with no ceiling—you can struggle for years without success, and then suddenly launch a billion-dollar business. It requires a different mentality.” (F11)*
Passion	*“But when an idea drives me like that, I mean, that excitement is my fuel. I can say I’ve filled the tank. I felt that kind of excitement.. I’ve been working for X for 7 years. Not a single day have I been unhappy.” (F8)* *“It covers every aspect, 24/7. And if something takes up your life 24/7, you have to be in love with it.. I love the work I do in every way. That’s why I do not see it as a job. It’s a part of my life.” (F9)*
Risk appetite	*“But when I look back, for example, when I attend a networking event, even if someone has not met me directly, if they have heard of X, they say, ‘Oh, was it the mother-daughter venture?’ So, in a way, we have created a personal brand value as mother and daughter, and we have directly reflected that into our company as two generations. Secondly, I think our risk-taking character is incredibly reflected in our companies…Because you take so many risks, at the end of the day, when you look at it, if this business does not work out, I feel like I will not exist either.” (F1)* *“My father-in-law is a contractor, and his family has always been involved in trade. He has a very high risk appetite. He can leave his dollar-earning job in America and try to start his own business in Turkey. But I’ve always been more of the mindset of evaluating what’s already in my hands. I’m someone who goes for more manageable risks. So, instead of high risks, low to medium risks appeal to me more. Of course, nowadays it’s no longer about unicorn startups, but rather the world of camel startups—companies that can survive on their own resources, like a camel that uses water wisely. We applied that methodology a bit ourselves.” (F11)*

### 4.4 Challenge and resilience

Participants report engaging in diverse and challenging tasks that utilize and expand their abilities. Challenges provide opportunities for building resilience, which associates with experiencing meaningfulness. Each participant has faced significant challenges in their respective fields, yet they have demonstrated persistence and adaptability in overcoming these obstacles. When they were insurmountable, they found a way out and looked somewhere else for moving on. Almost all of them refer to “uncertainty” as one of the biggest challenges that they had to learn to be comfortable with. The participants highlight access to funds as another formidable challenge. Psychological challenges seem secondary to financial obstacles and difficulties in access to funding. There are also regulatory challenges that they frequently voice out as primary reason for why they seek to go abroad, considering regulations are the hardest to affect. They take financial risks to realize their investment, and both idea creation and implementation might become their responsibilities. The resistance to change and introducing a new service/product with higher efficiency and persuading end users to replace their existing habits are also challenges entrepreneurs find as crucial obstacles to overcome before to score a significant and meaningful outcome. The (un)availability of a startup ecosystem that is not congenial to and welcoming of startup co-founders is yet another challenge. According to participants, team-making and dealing with team members’ problems might be challenging at times. Despite those challenges, the interviews reveal a common narrative of resilience among startup co-founders. Resilience and passion for entrepreneurial ideas/projects act as buffers to those challenges. Family is interestingly both a challenge to face at the initial stages of entrepreneurship and one of the strongest resilience factors throughout. Sample quotes are presented in Table 5.

**Table 5:** Quotes concerning the category “challenge and resilience.”

Codes	Quotes
Uncertainty	*“Do you know Sisyphus? He pushes a stone up a hill, and then it rolls back down from the top. He goes down and pushes it up again. He believes the gods are punishing him, and for eternity, he’s constantly pushing that stone up the hill, only for it to fall back down. It’s a bit like that. Just when you think, ‘I’ve learned it, I’ve succeeded,’ the story starts over again. For example, getting investment was a huge success for us in this period. Everyone celebrated it a lot. But it’s over now. We’ve started pushing the stone again. So, it’s a bit like this never-ending sense of not being fully satisfied, constantly being in the middle of a struggle.” (F9)* *“One of the mental lessons it teaches is about uncertainties. In reality, nothing in our lives is certain. We act as if things are certain and try to fit them into a mold.. Bro, I had set up this beautiful environment, pitched a tent, and I was lying down with the sun warming me up so nicely. It was a clear sky, and then a single cloud came and stopped right in front of the sun. I just laughed and thought, ‘This is it, no matter how much you try, that cloud is going to come and stay there.’” (F9)*
Financial challenges and access to funds	*“It’s like everyone has their own agenda. For example, I was really surprised—and I’m still surprised. They schedule meetings with me. We have the meeting, and there’s zero action, zero execution. Then I found out that they have meeting targets. So, they meet with me because they have to, not because they are going to invest in me. But while they are meeting with you, they say such nice things to keep you at the table. And you think, ‘Okay, they are probably going to invest.’ But they do not. Time wasted. Hours and hours. For me, it’s a loss. For them, it’s normal. Part of their job description. So, there was a disconnect there. That was the hardest part. It really gets on your nerves. It does, it really does. There were so many times where they promised to transfer the money. The investment, the contract, the signatures, everything was sent, and then they ghosted me. I still have one on my blacklist. I will never go back to them. After making so many promises, after taking all my financial history, all my data, everything, and then not even responding. I do not know, it just does not sit right. It does not fit human nature. And I’ve observed this a lot, unfortunately, in the investor world.” (F9)*
Regulatory and institutional challenges (startup ecosystem, resistance to change etc.)	*“I do not think it has to be that way. But there are regions where society acts more like a catalyst on this matter. It would work better if it were there. For example, when you are in San Francisco, once you walk out of a corporate company’s door—I’m creating a scenario right now—you grab a coffee and sit down. Then, a friend from Meta, Facebook, comes by. While you are talking about something with them, it’s likely that they would say, ‘Well, there’s actually a problem here, we could solve this.’ And 2 months later, you could join a program at Y Combinator there and test your startup idea.” (F2)* *“I worked on a project with X where they recreated Pompeii using augmented reality...I wanted to do the same for Varosha in Cyprus. In some way, they want to open the gates and restart tourism, reclaim the hotels, and there’s a huge story behind it. They could not open it before because the hotels were demanding compensation, saying, ‘We have not been able to use it for 50 years.’ One of my goals in Turkey was this. At some point, I wanted to start working on state projects and have the state behind me as a reference. I said, ‘Why do not you give me* Var*osha, and I’ll recreate the same project as Pompeii? I will not ask for a single penny, not even 1 TL. I’ll cover the entire project myself. I’ll show its old state, its current state, and the Eco-city version.’ (For Pompeii project), they are talking about 6 billion clicks! Varosha had terrible PR in the past. When you search for it now, you still see cursed things, like it’s a dangerous place, the war continues, etc. I said, ‘We can erase all of that, recreate everything from scratch, rebuild from zero, and I’m not asking for any money.’ But do you know we could not reach anyone for nearly 2 years? We tried Mersin Province Municipality, it did not work. We tried the Presidency, it did not work. This did not work, that did not work. We started experiencing this in many projects later on. For example, to bring change or for a state-related project, we proposed to Izmir’s Small and Medium Industry Development Organization to open laboratories in schools. We said we’d cover everything, provide the education in the labs, let the children start learning, developing projects, and building portfolios for themselves. But no response. Or you get stuck in some hierarchy.” (F4)* *“But they are not aware of it. I mean, we are creating a need from nothing, and for them, this could be the fastest version of a control process. But we are telling them that we are creating a need from nothing. It could be faster. Convincing people of this, especially when it’s a new technology, is a very difficult position to be in.” (F4)*
Psychological challenges	*“But I think, in a way, we have gotten used to it as part of our character. We’ve become people who motivate ourselves. Even when we struggle, we keep going somehow...And the fact that the person you started the journey with is still continuing makes you say, ‘Okay, one last run. Let us try this as well. If it does not work, we’ll reach that point.’...When you reach a point where you cannot find a way out of things, if there’s someone guiding you, you go and seek psychological support.” (F1)* *“It’s psychological. For example, I’m a very resilient person physically. I can wake up at 3 in the morning, go 3 days without sleep, and stand outside in the cold on a construction site. I’m that kind of person. But when it came to my mental state, I could not handle it. When I started the startup, I began experiencing physiological problems. I started seeing something like a light appear here on my head. Turns out it was psychological. It was something I’d never experienced before. I mean, my level of control over things is very high. In fact, I like stress. I enjoy being challenged. But psychologically, I could not quite manage it during that time.” (F7)*
Mismatch of expectations	*“When you take the service or product you are trying to solve to the market, sometimes the market’s demands do not quite match your expectations. Maybe you identified the needs in, say, June. But while the product is being developed, you can end up somewhat distanced from the market. When you bring the same service or product to the market 3 months later, you realize it does not exactly meet the market’s needs anymore. At this stage, you have also taken on investment. So, there are some commercial promises I’ve made to investors as well. Therefore, it requires taking very calculated steps.” (F2)*
Family	*“Then comes the pressure from the family, like, ‘Get a steady job already, have a career. Have a profession and stick to it. Be like everyone else, work as blue-collar or white-collar, have a stable income.’ Because in this, there’s no guaranteed income.” (F3)* *“Well, of course, it’s psychologically tough. It’s not easy. We do not always stay strong. There are moments when we cry, when we feel sad. It’s about self-motivation because no one from the outside says, ‘Hey, you can do it.’ In fact, when something is going really well, people usually do not say, ‘Yes, you are doing great, keep going.’ Instead, it’s more like, ‘Should I not be doing this?’ Even in the family, sometimes you hear, ‘My daughter, maybe you should find another job.’ I have not experienced it much personally, but I have friends who have been told, ‘Do not do a startup, quit it, and find something else.’ It’s an incredible roller coaster because you are taking risks.” (F12)*

### 4.5 Recognition and support

Startup co-founders find meaning in and through receiving constructive feedback and acknowledgment for their contribution. This might be in the form of social validation, attracting more investors and expansion to overseas. This theme emphasizes the importance of others’ appreciation and support for finding meaning in one’s initiatives. Within this theme, we can count customer feedback (verbal/online etc.), capital investment decisions, product promotions, social capital building activities including invitation to meetings, expeditions, among others. Participants engage in recognition and support building activities such as public opinion polls, experiments, product placements and promotions, discounted sales, etc. They report undertaking social responsibility projects such as providing mentorship to university students and offering gig work opportunities. Recognition and support also help co-founders find motivation and cultivate resilience to proceed through tough times. The importance of community and support systems is a recurring theme, highlighting the value of networks and collaborative effort. As such, it acts as complementary to challenge and resilience theme. Sample quotes are presented in Table 6.

**Table 6:** Quotes concerning the category “recognition and support.”

Codes	Quotes
Customer feedback and praise	*“For example, we do experiments on social media...We realized we went viral when we mentioned the ‘smart bra.’ Discoveries like this are actually what I enjoy. The trial-and-error, testing part is what keeps me going.”* (F1) *“Because as you achieve something, and as people start to notice you...As part of being an entrepreneur, we meet a lot of people, talk to a lot of people—professors, academics.. And maybe more people from the media.. As they talk more and validate you.. As they say, ‘Keep going with this, this has a lot of potential, you are doing great,’ these are very motivating words. It honestly satisfies you. That’s why we keep moving forward with even more motivation.”* (F3) *“And our AI said, ‘I’ve identified certain mistakes here. Let us highlight these products during these hours. By doing this, your sales will increase by 30%,’ it suggested. We implemented these recommendations for the businesses, and today we received a call where we found out that sales did not just increase by 30% but tripled. This made me incredibly happy. There’s a celebratory atmosphere in the company. Our AI gave the correct recommendation—more than correct, it exceeded our expectations. The feedback we have received shows this is going somewhere big. The excitement right now makes me very happy.” (F8)*
Expansion to new markets	*“I knew our product. I’ve used IBM. I’ve used SAP’s software. I’ve used all of them, and ours was by far the best. For example, we went and sold it to a bank in Germany, one of the biggest banks there. Turkish engineers selling technology to German engineers—that was something very meaningful to me. This, for instance, will keep me going for 2 years...By the way, we are currently managing 4% of Turkey’s energy. Right now, Apollo is in almost 1 out of every 25 businesses, and as a result, we have effectively planted over 10 million trees and achieved $35 million in savings. This makes us happy.” (F7)* *“For the past 4 years, we have put in a lot of effort into our AI model, and it’s significantly increasing sales for businesses. X is at a major turning point. We’re currently in 7 countries. We’ve established our company in the U.S. and are fully focused on the American market. I can say that when I wake up, all I think about is the U.S. market. Because there’s a huge gap there as well. During the 3 months I spent there, I clearly saw that digital menu companies were not focusing enough on the data side like we do. And we have made a huge deal with a major company.” (F8)* *“We established a company in the UK last year. We do not just want to be known in Turkey but globally as well. At least in the sports genetics testing market, yes, our goal is to be a pioneer in Turkey. But abroad, especially in the UK, if we can capture even 1% or 2% of the market, it would be great for us. Because it’s a rapidly growing sector, expected to reach $30 billion by 2030. If we can have a presence there, it would be amazing. As I said, if the algorithms are used on athletes and we see direct benefits, we can train the next generation of athletes or have a hand in that process, I believe that also creates social value. As I said, there’s both a commercial aspect and a social benefit aspect. If we can achieve both, we’ll have succeeded.” (F12)*
Product promotions, awards and prizes	*“It’s a job with no ceiling. You can work with the world’s largest companies with a very small team. And sometimes, there are moments when the other party’s recognition of you really moves the entire team, boosting morale and motivation. Because you are delivering a highly concentrated expertise with a small group, and you are developing a simple solution for a very complex problem. Since it’s somewhat related to engineering, my favorite part is when there’s a really complex problem. There are companies with thousands of developers and employees that cannot solve it. But you go in with your 10-person team, and you crack the problem. And in return, that company pays you...There’s a different world. Another world exists. And I have a chance to be a part of it. This world can open completely different doors for you, take you to completely different places. And I’m experiencing that right now. For example, we went to the U.S. with government incentives. Now, we are going to the UK. I’m involved in many processes related to the entrepreneurial ecosystem. Normally, if I asked for an appointment, they would not give it. So many people that I would not even find a spare minute to meet are now messaging me on LinkedIn saying, ‘Let us grab a coffee if you are at Kanyon (shopping mall in Istanbul).’ This is a really great thing.” (F11)*
Corporate responsibility projects	*“We have a friend working in the Presidency, and I learned that his mother is a primary school teacher in Antalya. In class, she asks the children a question: ‘If you could design and create a robot of your own, what would you want it to do in your life?’ One child says, ‘Crows come to my dad’s field and bother the crops, and my dad gets very tired and spends a lot of time dealing with them. I would want the robot to help chase away the crows from the field.’ This child explains how they envision positioning technology in their life based on a need. So, my friend’s mother asks, ‘Can we turn this into a nationwide movement in Turkey?’ The answer was, ‘Yes, we can.’ My friend then starts a movement called The Robot in My Dream. It became a highly impactful children’s movement, reaching primary schools from Edirne to Bitlis* (west to east provinces in Turkey). *Our goal was simple: we gathered teachers and told them to ask the same question to their students. Let the students design their robots. They could make it an assignment. Some could just draw it, others could create a matrix or build it with Legos. Record it on video. Even if they do not make anything, let them explain their dream robot. Record it on video, upload it to our channel, and tag us. We would feature the videos on The Robot in My Dream YouTube channel. We reached nearly 3,000 children. While we could not listen to all 3,000 stories, some outstanding stories started emerging, and teachers began sharing them with us. We did not expect it to reach this much impact.” (F10)*
Gigs	*“In X’s business model, there’s a crowdsourcing element, referred to as the X App. For example, right now, there are around 2,000 registered university students on the platform. The majority are studying in engineering departments, and most of them are in Istanbul. In X, there are some data processing tasks that cannot be automated, and these students are contributing manually to the AI, earning a good amount of income. Being able to provide this makes both my partner and me very happy because we were also students in Istanbul, and it’s financially tough there. So, when they tell us things like, ‘I labeled data for X and then bought myself a pair of headphones,’ it really means a lot to us…We try to provide mentorship to people from our school or other schools who are seeking help on this path, whenever we have time. We tell them that this job is not as fun and enjoyable as it might seem from the outside. You spend a lot of time on it for a very long period.” (F2)*

### 4.6 Work-life balance

This theme refers to co-founders’ experiences for managing their work-life balance, and their coping mechanisms, ways of managing stress, and finding customized strategies for maintaining work-life balance such as spending time with valued ones, travelling, gaming, exercising, reading, running, and others. The absence of a balance does not necessarily signal lack of happiness or satisfaction with life. Rather, most participants report being contend with their non-traditional (lack of) balance which probably opens opportunities for discovering their limits. This very absence of balance might even provide a meaningful experience. Their understanding of a balance might be crooked in favor of work but that does not exclude some fun or relaxing activities. We have recorded statements such as the following;

“We don’t really have set working hours. I mean, we can even be discussing things while having a coffee...I don’t have much of a work-life balance. But honestly, it’s not something that bothers me. I’m not bothered by it being this way.” (F1)

“Work-life balance and such concepts aren’t really a part of the lives of founders, to be honest.” (F2)

“There's no such thing as work-life balance, by the way...Someone once told me—'Do you believe in work-life balance?’ They said, ‘I believe in work-life harmony.’ Let me put it that way too.” (F7)

Sample quotes are presented in Table 7.

**Table 7:** Quotes concerning the category “work-life balance.”

Codes	Quotes
Stress coping activities	*“For example, I am someone who plays a lot of video games. Like, when I get bored, I turn it off and play a game.” (F1)* *“I set aside specific time for these things. I set aside specific time for my work. But all of them are things I love, you know. Like, I could get up and go, for example, I really love Formula 1. I could get myself a steering wheel.” (F4)* *“It’s definitely not a 9-to-5. Even if I go on vacation, I’m still working while on holiday. While my friends are swimming in the sea, at some point, I have to work. But at the end of the day, I also get to visit many countries and meet people I would not normally have the chance to meet.” (F12)*
Psychological counseling	*“I think every person.. This is not just because of work. It’s something that everyone needs to do in today’s world. Because.. We encounter so many manipulative things. In today’s world...social media is already a completely separate issue. Everyone in today’s world.. needs to get psychological support.” (F3)*
Exercising	*“Because, even though I say I feel free sometimes, a lot of crises emerge from every side. So, my brain needs to not deal with them for an hour. That’s why I’ve been working out a lot lately.” (F2)* *“Because the startup world is also about efficiency. It’s about being able to do a lot of work in a short amount of time, being multitaskers, or being self-learners. The ability to be a self-learner is important because in a startup, you do not have time to take a course. You need to learn quickly...But I still always make sure to structure my personal time, social life, and time for my spouse separately. For example, around 11:30, I close the computer and go to the gym. I do my workout for about 1–1.5 h. Then I sit down and open my computer again. In the evening, I have different hobbies that I spend time on accordingly.” (F11)*
Time with family and friends	*“I feel like there’s no difference between lying in my bed at home and lying on the couch in the office. Because of this, I’ve lost a lot of friends in my social life. For example, they say, ‘Let us go out,’ and I’m like, ‘Why do not you all come to our office, and we’ll have some wine there? I’ll check on my work in the meantime.’ I constantly try to turn my friend circle into a work environment.” (F5)* *“Very clearly, there’s no such balance. There’s family, there’s work. A third life, like a social life, does not exist. There’s no time for sports. I do not have a world where I can spend time for myself because 24 h is not enough. When I started this, I absolutely did not anticipate it would be this much.” (F6)*

Table 8 provides a summary of the above findings including overarching themes, main categories, and corresponding themes that we draw from interviews with co-founders regarding their perceptions of meaningfulness.

**Table 8:** Codes, corresponding categories and themes for co/founders’ perceived meaningfulness.

Overarching themes	Category	Codes
Meaningfulness professional	Significance	Positive personal impact, positive social impact, opportunity for growth and self-expression, making a difference, value added, trendsetting, original ideas
	Autonomy	Independent decision making, flexibility instead of strict hierarchical structuring, delegation of responsibility to the experts in the team, trustful relationships, moral licensing, providing space
Meaningfulness psychological	Identity	Self-work nexus, becoming one with work, startup as one’s baby, critical life experiences, values and beliefs, world view and life philosophy, passion, risk appetite
	Challenge and resilience	Challenges include uncertainty, financial challenges and access to funds, regulatory and institutional challenges (startup ecosystem, resistance to change etc.), psychological challenges, mismatch of expectations; family is both a challenge and resilience factor
	Work/life balance	Stress coping activities such as walking, exercising, gaming, travelling, camping, binge watching etc., psychological counseling, time with family, friends, and significant others
Meaningfulness social	Recognition and support	Customer feedback (verbal/online etc.), product promotions and praise, social capital building activities including invitation to meetings and expeditions, expansion to new markets, winning awards, and prizes, corporate responsibility projects and providing gigs for young people/university students

## 5 Discussion

Our goal in conducting this study was to provide insight into the dynamics of meaningful work in the context of entrepreneurship, particularly among co-founders of startups. The subjective perception that one’s work is important, meaningful, and contributes to a wider range of things is known as work meaningfulness (Rosso et al., 2010). It includes people’s opinions on the value of their work that extends beyond material benefits like the possibility for emancipation (Rindova et al., 2009). In accordance with this conceptualization, we contribute to a better understanding of how personal requirements and satisfaction of significance, autonomy, identity, and resilience intersect with the entrepreneurial experience by integrating the theoretical foundations of Self-Determination Theory (SDT) and the Job Characteristics Model (JCM). By utilizing a qualitative approach, we were able to capture the complex relationship between perceived meaningfulness and entrepreneurship, highlighting the distinctive combination of personal development, career advancement, and societal contribution that it provides.

**Figure 1:**
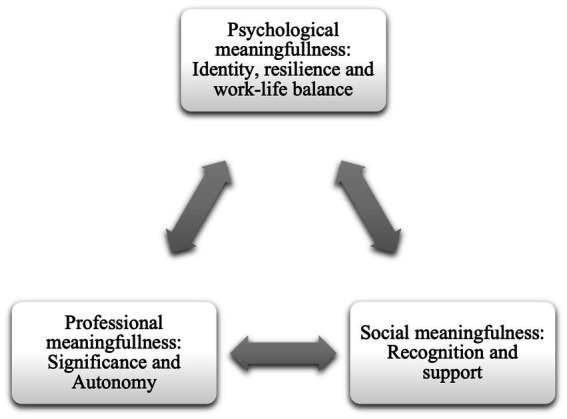
A tripartite model of entrepreneurial meaningfulness.

Utilizing the premises of both Self Determination Theory and Job Characteristics Model in our study, we propose a multidimensional model of work meaningfulness comprising significance, autonomy, identity, challenge and resilience, recognition and support, and work-life balance. Significance refers to the belief that one’s work matters and has a positive impact on others or society. Research has found perceptions of significance to be a determinant of job satisfaction (Hytti et al., 2013). Autonomy represents the degree to which individuals perceive to have control over their work processes and decisions with previous research documenting higher levels of satisfaction among self-employed people due mostly to their greater levels of autonomy (Benz and Frey, 2008) and research investigating the intricate dynamics of autonomy in entrepreneurship (van Gelderen, 2016). Identity refers to the alignment between one’s work and personal values, beliefs, and sense of self. Previous research shows that a sense of identity is central to entrepreneurial processes (Malmström, 2021; Shepherd and Haynie, 2009). Challenge and resilience describe entrepreneurs’ experiences of engaging in diverse and challenging tasks that utilize and expand their abilities, with research exploring the role of challenges and resilience during entrepreneurship journey (Brieger et al., 2021; Bullough and Renko, 2013; Shir et al., 2019). Previous research has acknowledged the role that challenges play in constructing meaningful work experiences, such as Bailey and Madden (2016) who claimed that “*the experience of coping with these challenging conditions led to a sense of meaningfulness far greater than they would have experienced dealing with straightforward, everyday situations*.” This is in line with the premises of the job characteristic model associating jobs that allow for some experiences such as greater skill variety and significance to be seen as more meaningful. Recognition and support contribute to perceived meaningfulness through receiving constructive feedback and acknowledgment for one’s contributions. Research has investigated the role of feedback in performing an entrepreneurial task (Haynie et al., 2012). Work-life balance, achieving a satisfying level of equilibrium between the demands of work and life with positive experiences across both domains, associates with higher levels of entrepreneurial subjective well-being which in turn has an indirect impact on firm growth (Drnovšek et al., 2024). Work-life balance is crucial for entrepreneurs, who often blur the lines between work and personal life. Positive experiences in balancing these domains lead to enhanced well-being and productivity. Previous research provide evidence that work centrality and the degree of involvement with one’s work compared to other domains of life will likely to be associated with higher levels of meaningfulness, and individuals with high levels of work centrality will probably find job loss and retirement to be almost devastating experiences (Rosso et al., 2010). Collectively, these themes and appertaining codes comprise our multidimensional model of perceived meaningfulness for startup co-founders (see Figure 1 for a summary).

Our findings suggest that the fulfillment of intrinsic and extrinsic needs plays a pivotal role in shaping entrepreneurs’ sense of purpose. The ability to exercise creative control and the challenge of navigating uncertainty are highlighted as key motivators. Entrepreneurs derive meaning not only from the freedom to pursue their vision but also from the resilience developed in response to the inevitable hurdles they face (Bullough and Renko, 2013). This aligns with the JCM’s premise that high autonomy and task significance foster motivation and engagement (Rauch and Frese, 2007). Hackman and Oldham’s contention that experienced meaningfulness of work is a key psychological condition for development of internal work motivation applies to our sample co-founders as well.

Moreover, in line with the premises of self-determination theory which posits that experiences of autonomy, competence and relatedness lead to motivation, and enhanced levels of motivation result in greater levels of experienced meaningfulness, we find that co-founders derive experienced meaningfulness in professional, psychological, and social domains. As such, our study extends the conversation beyond individual motivations by examining how social recognition and identity contribute to entrepreneurial meaning. Entrepreneurs frequently intertwine their personal values and professional endeavors, positioning their ventures as extensions of their own identity (Shepherd and Haynie, 2009). This reflects a deeper existential pursuit, where work transcends economic gain and becomes a vehicle for self-expression and societal impact. Recognition from peers, customers, and the broader community further amplifies this sense of purpose, echoing findings that social validation enhances job satisfaction and resilience (Brieger et al., 2021).

It should be noted however that entrepreneurial meaningfulness is not a distinct type of meaningfulness but rather a contextualized experience of meaningful work shaped by the entrepreneurial journey. This concept captures how entrepreneurs derive meaning through personal alignment with their ventures, resilience in overcoming challenges, and contributions to broader societal goals. For example, participants frequently linked their sense of significance to the impact of their work on society, such as addressing environmental challenges or fostering community development. Similarly, autonomy emerged as a cornerstone of entrepreneurial meaningfulness, allowing participants to align their work with personal values and to exercise creative control. However, autonomy was also constrained by external factors, such as investor demands, highlighting the tensions inherent in the entrepreneurial process. Identity alignment played a critical role, with participants often describing their ventures as extensions of themselves—a perspective that blurred the lines between personal and professional identities. Despite, our study contributes to the literature by shifting the focus from static models of meaningful work to a more dynamic understanding of how meaning evolves in response to entrepreneurial challenges and opportunities.

This tripartite model reflects a holistic understanding of how entrepreneurs derive meaning from their work, with psychological resilience, professional mastery, and societal contribution as interlocking components. The model allows for a dynamic interpretation of meaningful work, where these dimensions interact fluidly, compensating for weaknesses in one area by drawing on strengths in another. Psychological dimension, consisting of identity, resilience, and work-life balance, represents aspects that are central to how entrepreneurs navigate the uncertainty of startup ventures. Identity is deeply entwined with their work, as entrepreneurs often view their ventures as an extension of themselves, blurring the lines between personal and professional identities. Previous research has revealed that founders are seeking to align their personal identity with that of a founder’s identity through the life cycle of their venture (O’Neil et al., 2022). Resilience plays a crucial role in how these entrepreneurs withstand challenges. They often frame their setbacks as part of a broader narrative of growth and personal development, which aligns with self-concept theories. As such, the psychological experience of meaningfulness is highly personal, shaped by intrinsic motivations and the sense of overcoming obstacles through resilience. Professional dimension, consisting of significance and autonomy, emphasizes how entrepreneurs derive meaning through the significance of their work and enjoyment of freedom in decision-making and direction-setting. For many, receiving feedback, whether from customers, investors, or team members, reinforces their sense of competence and task significance. However, the role of feedback should be evaluated with caution given the potential for being overwhelmed by an excessive amount of it (Baur et al., 2024). At a societal level, meaningful work is often tied to the broader social or environmental impact entrepreneurs aim to achieve. For instance, participants in the study reference their drive to contribute to sustainability or societal wellbeing, aligning their ventures with global challenges such as environmental conservation or social equity. This dimension is particularly reflective of eudaimonic well-being—the pursuit of meaning through contribution to a greater good (Hahn et al., 2012; Ryff, 2019; Shir and Ryff, 2022). Entrepreneurship is not merely about personal or financial gain but is often perceived as an avenue to create value for society, communities, or specific social causes. That is one reason why the Corporate Social Responsibility lens could also be applied to understand how entrepreneurial activities are increasingly tied to ethical considerations and societal responsibilities, enhancing the perceived meaningfulness of the work (Rosário and Figueiredo, 2024).

In line with the previous research from different contexts and using different approaches (Tommasi et al., 2024), we utilized The Job Characteristics Model and Self-Determination Theory as theoretical backbones, to explain why autonomy, significance and feedback are so crucial to the entrepreneurial experience of meaningful work. The autonomy enjoyed by entrepreneurs allows for greater alignment between personal values and work, contributing to both professional satisfaction and personal fulfillment. Simultaneously, the challenges inherent in entrepreneurship—such as financial instability or market uncertainties—are mitigated by resilience-building, which is often fostered through professional networks, social validation, and community support. For startup founders, who often wear multiple hats and engage in diverse activities, the professional challenges they face enhance their sense of job fulfillment, provided they can demonstrate competence and receive validation through positive outcomes. The integration of SDT and JCM in this study reveals that the entrepreneurial journey, while fraught with challenges, provides fertile ground for meaningful work. The entrepreneurial context offers unique opportunities for individuals to actualize their potential, exercise autonomy, and contribute to broader societal goals. Two overarching theories provide justification for our tripartite model while our model goes beyond the dominantly psychological perspective of existing organizational research on experienced meaningfulness.

Finally, we conducted the current study in Turkiye, an emerging country with a predominantly risk averse cultural context. With an uncertainty avoidance score of 85 and power distance score of 66 out of 100 in terms of Hofstede’s culture typology (The Culture Factor Group, 2024), Turkiye’s culture still displays a hierarchical predisposition and overall tendency to avoid risky initiatives associated with entrepreneurship. From this lens, an entrepreneurial risk appetite runs counter to the general cultural values in Turkiye. Previous research has associated the alignment between social and cultural values (such as individualism versus collectivism), and those of the individual to be a source of motivation for entrepreneurship and perceived meaningfulness (Assmann and Ehrl, 2021; Rosso et al., 2010). As such, we could have identified different meaningfulness experiences in our study than those reported in WEIRD contexts. However, one of the largest research initiatives on meanings of work on a sample of 15,000 participants across eight different countries has interestingly shown that meanings of work across cultures were “more alike than different,” with similar levels of work centrality and intrinsic values (MOW International Research Team, 1987). It seems that co-founders across the digitalized world becomes increasingly similar in their experienced meaningfulness, and it is highly likely that there is more within-culture variety in terms of entrepreneurs’ meaningfulness experiences than between cultures. However, more research is needed from the emerging world for more nuanced discussion of such arguments.

## 6 Implications for practice

On the psychological dimension, entrepreneurs often experience high levels of stress due to the inherent uncertainty of startups. Practical initiatives such as mental health programs, peer mentorship, and coaching services could be implemented to help entrepreneurs build psychological resilience. These initiatives should focus on helping them navigate setbacks and view challenges as learning opportunities, enhancing both their personal growth and business outcomes. Given that autonomy and identity alignment provide a large amount of meaning to entrepreneurs, support networks that maintain this autonomy are essential components of entrepreneurial ecosystems. Business incubators, accelerators, and investors should ensure that while offering guidance, they do not undermine the entrepreneurial freedom to innovate and steer their ventures in line with personal values.

On the professional dimension, targeted training programs designed around key entrepreneurial skills—such as leadership, financial management, marketing, and innovation—can enhance their sense of competence. Additionally, programs must be designed to offer ongoing feedback, as this is crucial for preserving motivation and job engagement. One of the most important things for an entrepreneur to feel satisfied in their job is validation. This may be achieved through structured feedback loops, which include peer-based appraisal, customer reviews, and frequent check-ins with investors.

On the social dimension, entrepreneurs who perceive their work as socially valuable experience higher levels of meaningfulness and engagement. Embedding social impact into business models—through Corporate Social Responsibility strategies, sustainability initiatives, or addressing local community issues—can provide entrepreneurs with a greater sense of purpose. Many entrepreneurs aim to solve societal challenges, from sustainability to social equity. By offering rewards to businesses that emphasize social innovation—like subsidies or public-private partnerships—policymakers and other stakeholders in the business ecosystem can promote this approach. This could make socially conscious entrepreneurship more visible and feasible, facilitating the integration of social objectives with financial success for business owners.

## 7 Limitations and future directions

Investigating work meaningfulness leads to a better comprehension of people’s desire to find significance in their work. We would like to draw attention to a few areas that warrant further investigation.

Longitudinal designs that monitor the evolution of entrepreneurs’ perceptions of meaningful work could be advantageous for future research. Researchers can gain a better understanding of how the psychological, professional, and societal aspects of work meaningfulness fluctuate and how these fluctuations affect both long-term business success and subjective well-being by studying entrepreneurial teams at various stages of their ventures (e.g., early-stage, growth, exit) (Patzelt et al., 2021; Strese et al., 2018). In a similar vein, previous research that has compared experienced entrepreneurs with novice entrepreneurs has shown marked differences between their perceptions and approaches (Baron and Ensley, 2006).

Entrepreneurship takes place in different cultural contexts, each with its own set of norms and values (Pinillos and Reyes, 2011; Tiessen, 1997). Research should explore how the Tripartite Model of meaningful work applies across various cultural settings. For instance, do entrepreneurs in individualistic cultures (focused on autonomy and self-expression) derive meaning differently than those in collectivist cultures (focused on community and societal contributions)?

Research could investigate potential gender differences in how entrepreneurs perceive and construct meaning in their work. Previous studies suggest that women entrepreneurs might prioritize relationship-building and societal impact more strongly than their male counterparts (Fernández-Guadaño and Martín-López, 2023). Understanding these gendered experiences can provide a more nuanced picture of how men and women derive meaning from entrepreneurial ventures, particularly in areas like work-life balance and social contribution.

In addition, we acknowledge that demographic characteristics might play roles in meaningful work experiences. Although age has been related with declining levels of wellbeing, there is the possibility that entrepreneurial activities in later ages could offset the later-age wellbeing declines by providing purpose. Temporality of meaningful work experiences has already been documented by Bailey and Madden (2017) who displayed the relatively episodic nature of this experience across different occupations, and this could have repercussion on an intraindividual level where different life stages could associate with different degrees of meaningfulness perceptions. This line of research has recently gained momentum, differentiating between meaningfulness experiences as either a permanent/steady mindset or as a changeable/episodic experience, with repercussions for the digital world of work (Tommasi et al., 2020). Positive interpersonal relationships and family life experiences have been found to contribute to higher levels of wellbeing through helping behaviors and personal growth opportunities (Ryff, 2019). Entrepreneurial ventures might also contribute to and be influenced by high quality interpersonal relationships and family roles. In addition to many other benefits, entrepreneurial activities have the potential to promote and enhance individual and societal wellbeing, on both physical and psychological grounds, which stands as a relatively underexplored venue especially in nascent emerging country contexts.

Despite its potential contributions to research and practice, our study has several limitations. First, although our participants are currently active in the largest cities in the country, the sample size remains relatively small, limiting the generalizability of our findings to the broader startup ecosystem within the country or similar contexts globally. Furthermore, a more stratified sample, encompassing startups at various stages of their lifecycle—early, mid, and late—could offer deeper insights into how experiences of meaningfulness evolve over time. Lastly, expanding the participant pool to include startups distributed across all seven geographic regions would provide a more comprehensive and geographically diverse perspective on our topic of interest. Last but not the least, our study relies on self-reported data, which is subject to biases such as social desirability or retrospective inaccuracies, potentially affecting the reliability of our findings. Future research could address these limitations to enhance the robustness and applicability of the insights.

## Data Availability

The raw data supporting the conclusions of this article will be made available by the authors, without undue reservation.
